# Extracts

**Published:** 1884-05

**Authors:** A. V. Meigs

**Affiliations:** Physician to the Pennsylvania Hospital and Children’s Hospital of Philadelphia


### EXTRACTS

From an article on food for infants by A. V. Meigs, M.D., Physician to the Pennsylvania Hospital and Children's Hospital of Philadelphia.

The necessary data being at hand, it is comparatively easy to construct a food which shall, at least be more nearly what is needed than any previous one. In making such a food, there are two matters to be considered : the proximate constituents must be in the same relative proportions as they are found in human milk, and they must be in a medium which shall be, as human milk is, alkaline. This latter end is easily accomplished by the use of a due amount of lime-water, and is justified by the fact that it is a matter of experience, almost universally acknowledged as true, that it is a most useful adjunct, rendering cows' milk more easy of digestion by the human stomach. The quantity of lime-water to be used should be one-fourth of the total by measure. This may seem to many persons an excessive quantity; but when it is understood that if made as ordinarily directed, by agitating water with lime and then filtering, it contains only two decigrammes of lime in each litre, it becomes plain that the use of lime-water means the administration of a great deal of water and very little solid matter. The above estimate was arrived at by direct experiment; 10 cc. of freshly made, filtered lime-water, being evaporated, was found to yield two milligrammes of lime. This is a very large estimate of the amount of lime soluble in water, as may be seen by reference to the United States Dispensatory, or the National Dispensatory, both giving it as much less. That the use of lime-water (alkali) in an infant food makes a difference in its behavior with some reagents is shown by the following experiments. A food was made in the proportions which will presently be given, and 10 cc. of it agitated with ether and alcohol, as directed in my previous paper read before the Society, for the extraction of the fat; it was found that the coagulation took place in the form of a fine network, which remained permanently distributed through the lower stratum of the liquid, no sediment forming at the bottom. When an exactly similar mixture was made, except that the lime-water was replaced with water, leaving the fluid acid, and this agitated with ether and alcohol, thick, heavy curds formed, which at once sank to the bottom. Again, when two mixtures--one with and the other without lime-water--were treated with ten drops of acetic acid, the one without lime-water showed much larger, heavier coagulæ than that which contained lime-water. These experiments show with certainty that the addition of lime-water does alter the coagulability of the casein when experimented with, whatever may take place in the stomach; and I have already quoted Lehman's opinion that the acidity or alkalinity of milk makes a difference in the formation of the coagulum. Whatever may be the value of these artificial experiments, the great reason for the use of lime-water is that the experience of man has found it good, and that is sufficient reason for its use in the present state of knowledge. It is quite possible that in the future something better may be found—phosphate of lime perhaps, for it is the salt which exists in milk in larger quantity than any other; but further and exhaustive study of the inorganic constituents of both human and cows' milk will be required to place this matter upon an exact scientific basis. It is very desirable that further study of the salts of milk should be prosecuted, and it is much to be hoped that in the near future exhaustive analyses will be made. The amount of inorganic matter in cows' milk is so much greater than that in human milk that, as there is at present no means of removing it without altering or destroying the other component parts, no infant food can be made exactly like human milk in respect to the amount of salts contained.

So far as bringing the other proximate constituents to like proportions with those in human milk, the first step must be to so dilute with water as to get the desired quantity of casein; the fat and sugar can be increased by the use of the necessary quantities of cream and commercial milk-sugar. Taking the averages of cream and good city milk as already given, it will be found by calculation that if there be mixed together 10 cc. of cream, 5 cc. of milk, 10 cc. of lime-water, and 15 cc. of water, with 2.2 grammes of milk-sugar, the desired mixture is had. That this is the case should not be trusted to mere calculation, but an analysis of the mixture should be made, both to verify the calculation and to observe how the mixture behaves when subjected to the analytic processes, whether it in its reactions more closely resembles cows' milk, with which it is made, or human milk.

The easiest way to prepare and use the food is as follows: There must be obtained from a reliable druggist packages of pure milk-sugar containing seventeen and three-quarters drachms each. The contents of one package is to be dissolved in a pint of hot water, and it is best to have a bottle which will contain just one pint, as there is then no need for further measuring. The contents of one of the sugar packages is put into the bottle, and when filled with hot water the sugar soon dissolves, and it is ready for use. The dry sugar keeps indefinitely, but after it is once dissolved it sours if kept more than a day or two in warm weather; it is understood, therefore, that the sugar-water must be kept in a cool place, and if it should at any time become sour, as is easily discovered if it is smelled and tasted, it should be thrown out, and after the bottle has been carefully washed with boiling water, the contents of a fresh package dissolved. A milk-man must be found who will serve every day fresh, good milk and cream. By good milk is meant ordinary milk, such as is easily procured in most cities, and not rich Jersey milk; and in the same way the cream should be such as is ordinarily used in tea and coffee, and not the very rich cream of fancy cattle. The reason that ordinary milk and cream are recommended, is because they are within the reach of almost every one, and not because they are any better than the rich milk of high-bred stock. If Jersey milk was to be used, it would be necessary to analyze specimens, and then make the necessary calculations as to how to dilute it to obtain the desired relative proportions of the proximate principles. When the child is to be fed, the nurse should mix together two tablespoonfuls of cream, one of milk, two of lime-water, and three of the sugar-water, and then as soon as the mixture has been warmed it may be poured into the bottle, and the food is ready for use. If the infant is healthy this quantity will not satisfy it after the first few weeks, and then double the quantity must be prepared for each feeding. Twice as many tablespoonfuls of each of the ingredients must be mixed together, making sixteen tablespoonfuls (about half a pint) in all.

This food should not be given any stronger until the child is eight or nine months old at least, but if the infant is a healthy one, it may take as much of it as it wants, but always of the same strength. A robust infant will often take three pints, or even more, in each twenty-four hours. It is an easy matter for any one to learn how to make the lime-water; and it is advisable to have it made at home, for a great deal is used, and if it is made at home much trouble and expense are saved.

With regard to the propriety of increasing, from week to week, the strength of any artificial food given to infants, there has been some question. Most authorities have advised that the foods should be increased in concentration until finally the infant is given pure cows' milk. The propriety of this procedure, during the earlier months of life at any rate, is very doubtful. Although there is some reason to believe that the quantity of solid ingredients in human milk increases from month to month as lactation goes on, such an opinion should be accepted only with great caution, for it seems likely that if there is any increase in the concentration of the milk after the colostrum has once disappeared, and the nursing process has settled down into its even course, the increase is so slight that it may be disregarded. Analyses show that the milk of a woman whose child is two months old does not differ materially from that of one whose child is twelve or fifteen months old. If, then, nature has made no difference, which our means of analysis will detect, between the milk of a woman who has been nursing two months, and one who has nursed twelve, an artificial food which has been found to suit an infant of two months, should be made more concentrated only very gradually, and with careful observation of the effect upon the health of the infant. It is best, therefore, if the infant thrives and grows as it should, not to make any change in the food until after six to nine months of age have been attained.

With regard to the use of condensed milk as a food for young infants, I can only repeat what I said in my former paper, that I cannot believe that any article which has been canned, and kept for weeks or months, or perhaps still longer, can be so good as the same thing when fresh. My table of analysis shows the composition of the dilution of condensed milk commonly used in this city, and it shows that the proportion of fat is much too small, and for this reason, partly at least, it fails as a food. Its success is due to the fact that it contains nearly the same proportions of casein and sugar as exists in human milk. Dr. Ellwood Wilson is in the habit of directing that after the first few weeks a small proportion of fresh cream be added to the condensed milk, and this would render it still more nearly what is needed; this practice, which cannot be too much commended, if condensed milk is used, is not, however, at all a common one. Withall, I am unable to believe that condensed can be as good as fresh milk, if properly used. There are many other points of great interest, connected with this subject, which I should have liked to bring to your attention, but my paper has been already much too long.

